# Lesion Length Improves Diagnostic Accuracy of Intravascular Ultrasound for Detecting Functional Intermediate Coronary Stenosis Evaluated With Coronary Angiography-Derived Fractional Flow Reserve in Non-left Main Artery

**DOI:** 10.3389/fcvm.2021.715514

**Published:** 2021-09-30

**Authors:** Menghuan Li, Iokfai Cheang, Yuan He, Shengen Liao, Hui Wang, Xiangqing Kong

**Affiliations:** ^1^Department of Cardiology, The First Affiliated Hospital of Nanjing Medical University, Nanjing, China; ^2^Gusu School, Nanjing Medical University, Suzhou, China

**Keywords:** intravascular ultrasound, computational angiography-derived fractional flow reserve, intermediate coronary artery stenosis, three dimensional quantitative coronary angiography, diagnostic accuracy

## Abstract

**Objective:** Intravascular ultrasound (IVUS) parameters, for example, minimal lumen area (MLA) and area stenosis (AS), poorly identified functional intermediate coronary stenosis (ICS). For detecting functional ICS defined by coronary angiography-derived fractional flow reserve (caFFR), our study aims to determine whether IVUS parameters integrated with lesion length (LL) by three-dimensional quantitative coronary analysis (3D-QCA) could improve diagnostic value.

**Methods:** A total of 111 patients with 122 ICS lesions in the non-left main artery were enrolled. MLA and AS were calculated in all lesions by IVUS. Diameter stenosis (DS%) and LL were measured by 3D-QCA. caFFR was computed by the proprietary fluid dynamic algorithm, a caFFR ≤ 0.8 was considered as functional stenosis. Receiver-operating curve analyses were used to compare the diagnostic accuracy among indices to predict functional stenoses.

**Results:** Mean caFFR values in all lesions were 0.86 ± 0.09. Lesions with caFFR ≤ 0.8 showed lower MLA and higher AS (MLA: 3.3 ± 0.8 vs. 4.1 ± 1.2, *P* = 0.002; AS: 71.3 ± 9.6% vs. 63.5 ± 1.3%, *P* = 0.007). DS% and LL were more severe in lesions with caFFR ≤ 0.8 (DS%: 45.5 ± 9.6% vs. 35.5 ± 8.2%, *P* < 0.001; LL: 31.6 ± 12.9 vs. 21.0 ± 12.8, *P* < 0.001). caFFR were correlated with MLA, AS, and LL (MLA: *r* = 0.36, *P* < 0.001; AS: *r* = −0.36, *P* < 0.001; LL: *r* = −0.41, *P* < 0.001). Moreover, a multiple linear regression analysis demonstrated that MLA (β = 0.218, *P* = 0.013), AS (β = −0.197, *P* = 0.029), and LL (β = −0.306, *P* > 0.001) contributed significantly to the variation in caFFR. The best cutoff value of MLA, AS, and LL for predicting caFFR ≤ 0.8 were 3.6 mm^2^, 73%, and 26 mm, with area under the curve (AUC) of 0.714, 0.688, and 0.767, respectively. Combined with MLA, AS, and LL for identifying functional ICS, the accuracy was the highest among study methods (AUC: 0.845, *P* < 0.001), and was significantly higher than each single method (All *P* < 0.05).

**Conclusion:** Lesion length can improve the diagnostic accuracy of IVUS-derived parameters for detecting functional ICS.

## Instructions

Intermediate coronary artery lesion refers to stenoses with 40–70% of stenosis severity found by coronary angiography (1). Currently, percutaneous coronary intervention (PCI) is one of the most common invasive therapeutic techniques worldwide, showing a tremendous survival benefit in patients with acute coronary syndromes and patients with significant stenosis deemed myocardial ischemia (2–4). However, the decision of revascularization or medical therapy remains controversial in patients with intermediate coronary stenosis (ICS), in which the optimal assessment method of these functional significances was essential.

Functional assessment was essential to explore myocardial perfusion for ICS lesion. Fractional flow reserve (FFR) is a validated index for evaluating functional severity of coronary artery lesions (3, 5). FFR < 0.8 is usually considered as a positive functional stenosis, which indicates myocardial ischemia. Functional assessment of ICS by FFR was shown to be superior to visual assessment for therapeutic strategy-making (5). However, the use of invasive pressure wire and induction of hyperemia are required during the measurement of FFR, causing patient discomfort and limiting its clinical use (6). In recent years, computational approaches-derived FFR indices have been developed to detect functional coronary stenosis noninvasively. FLASH FFR study showed good diagnostic performance of a novel coronary angiography-derived FFR (caFFR), with a diagnostic accuracy of 95.7%, sensitivity of 90.4%, specificity of 98.6%, and the area under the curve of 0.979, to detect an invasive FFR ≤ 0.8 (7).

Intravascular ultrasound (IVUS) is a common method to evaluate functional intermediate stenosis in clinical practice. However, several studies demonstrated that minimal lumen area (MLA) and area stenosis (AS) had a weak-moderate correlation with FFR (1, 8–10). In addition, the best threshold of IVUS-MLA or IVUS-AS for detecting functional ICS remains controversial. The accuracy of identifying functional stenosis by IVUS parameters was not satisfied compared with FFR.

A previous study reported that lesion length (LL) calculated with quantitative coronary angiography (QCA) was associated with myocardial ischemia (11). In this study, we hypothesized that integrating LL could improve accuracy for detecting functional ICS. Our study aims to determine whether IVUS parameters, including MLA and AS, integrated with LL measured by three-dimensional QCA (3D-QCA) could improve diagnostic value for detecting caFFR ≤ 0.8.

## Methods

### Study Population

This observational retrospective single-center study enrolled patients with ICS who underwent IVUS evaluation from January 2014 to January 2019. ICS was defined as 40–70% diameter stenosis on visual estimation. Exclude criteria were: left main coronary artery (LMCA) lesions, bypass graft lesions, true bifurcation lesions, poor quality of coronary angiogram precluding caFFR computation (e.g., substantial foreshortening or overlap of the vessels, absence of two angiographic projections with the view of at least 30° apart, insufficient contrast flush), ostial lesions in a major artery, and incomplete data of IVUS parameters.

Study protocol was approved by the independent institutional ethical committee of the First Affiliated Hospital of Nanjing Medical University. The study complied with the Declaration of Helsinki. Written informed consents were obtained from all participants before examinations.

### IVUS Imaging

IVUS imaging was performed after intracoronary administration of 200 ug nitroglycerin using a 20-MHz phased-array transducer (Eagle Eye Gold^TM^) coupled with an imaging console (Volcano, Rancho Cordova, CA). The transducer was introduced to the distal portion of target lesions and was pulled back to the proximal vessel at a speed of 0.5–1.0 mm/s. MLA was analyzed at the site of most stenosis with the smallest lumen area. The reference vessel cross-sectional area was measured by identifying the edge of the adventitia. AS was calculated as reference cross-sectional area minus MLA and divided by reference cross-sectional area.

### caFFR Measurement

The operator who performed caFFR computation (Rainmed Ltd., Suzhou, China) was blind to IVUS results. To assess caFFR, two angiographic images of the target vessel excluding overlapped or foreshortening vessels, separated by at least 30° apart were selected to reconstruct a three-dimension (3D) model of the coronary artery. The model provided 3D-QCA data, including LL, diameter stenosis fraction (DS%), and flow speed. Invasive aortic blood pressure was reviewed from the data of the institutional catheter center and was input to the FlashAngio console. Resting flow velocities were determined by TIMI Frame Count and anatomical information was derived from the 3D model. caFFR were calculated by FlashAngio software with a proprietary fluid dynamic algorithm. Similar to FFR, a caFFR of ≤0.8 was considered significant.

### Statistical Analysis

Data distribution was assessed by the Kolgormonov-Smirnov test. Numerical variables with normal distribution were presented as the mean ± standard deviation and were compared by independent sample student's *t*-test. Non-normally distributed continuous variables were presented as median with interquartile range (IQR) and compared by non-parametric Mann–Whitney *U* test. Categorical variables were calculated using counts and percentages, and were evaluated by Chi-square test or Fisher exact test as appropriate. The correlation between caFFR and MLA, AS, and LL was analyzed by calculating Pearson R correlation coefficients for variables with normal distribution or Spearman rank's correlation coefficients for variables with non-normal distribution. Linear regression analysis was conducted to evaluate the effect of MLA, AS, and LL on caFFR. Receiver operation curves (ROC) analysis was applied to assess the best cutoff values of MLA, AS, and LL predicting caFFR ≤ 0.8 using MedCalc (version 18.2.1, Mariakerke, Belgium). The best cutoff value was calculated using the Youden index. Sensitivity, specificity, positive predictive value, and negative predictive value with 95% confidence intervals were determined for each cutoff value. The combined diagnostic power of MLA, AS, and LL was estimated with ROC by adding a new variable calculating by binary logistic regression. *P* < 0.05 was considered statistically significant. Statistical analyses were performed by Statistical Program for Social Sciences 21.0 software (SPSS, Inc., Chicago, IL).

## Results

**Figure 1:**
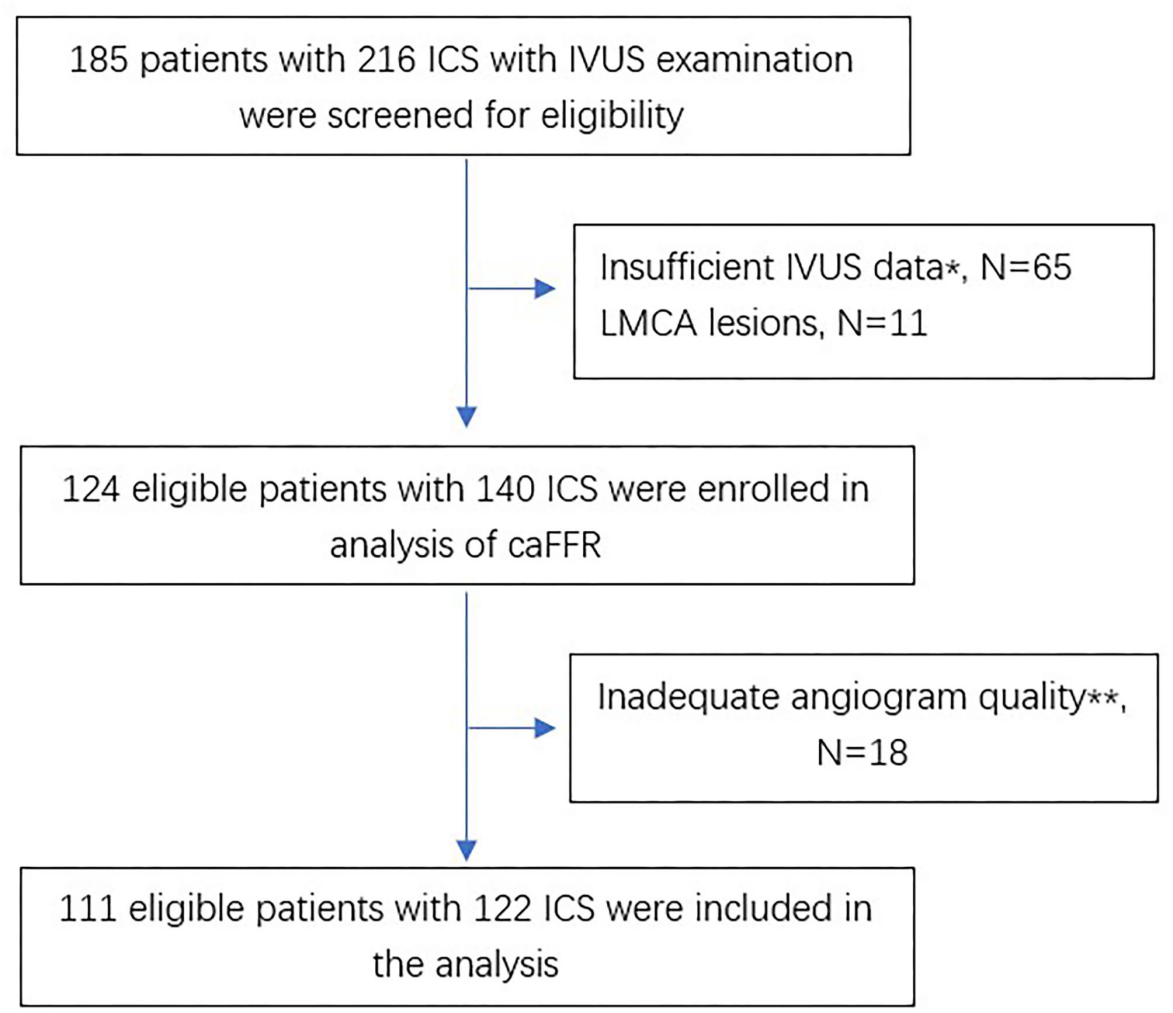
Flow chart of the study. ^*^Absence of any of MLA or AS. ^**^Including substantial foreshortening or overlap of the vessels, absence of two angiographic projection with the view of at least 30° apart, insufficient contrast flush, ostial lesions in major artery. ICS, intermediate coronary stenosis; IVUS, intravascular ultrasounds; LMCA, left main coronary artery; caFFR, computational pressure-flow dynamics derived fractional flow reserve.

Figure 1 shows the flow chart of this study. A total of 185 patients with 216 ICS lesions assessed by IVUS were screened for eligibility. There were 140 lesions enrolled in the analysis of caFFR, of which, 111 patients with 122 ICS lesions completed 3D-QCA and caFFR analysis. The main reasons for screening failure were insufficient IVUS data (65 lesions, 30.1%), LMCA lesions (11 lesions, 5.1%), and inadequate angiogram quality (18 lesions, 8.3%).

Clinical characteristics of patients are presented in Table 1. Mean age was 63.6 ± 10.4 years, 71.2% were male. Risk factors including hypertension, diabetes mellitus, hyperlipidemia, and smoking had no significant differences between the caFFR ≤ 0.8 group and the caFFR > 0.8 group; while stroke was more frequent in patients with caFFR ≤ 0.8. Forty patients were diagnosed as stable angina pectoris (SAP), 59 as unstable angina pectoris (UAP), nine as ST-segment elevated myocardial infarction (STEMI), and three as non-ST-segment elevated myocardial infarction (NSTEMI). No significant difference of distribution among the presentation of patients was shown between groups.

**Table 1:** Clinical characteristics of patients between the caFFR ≤ 0.8 group and the caFFR > 0.8 group.

**Patient characteristics**	**All*N* = 111**	**caFFR ≤ 0.8 *N* = 22**	**caFFR > 0.8 *N* = 89**	***P* value**
Age (years)	63.61 ± 0.4	64.31 ± 0.0	63.51 ± 0.6	0.755
Male	79 (71.2)	16 (72.7)	63 (70.8)	0.857
Hypertension	69 (62.2)	16 (72.7)	53 (59.6)	0.254
Diabetes mellitus	24 (21.6)	6 (27.3)	18 (20.3)	0.472
Hyperlipidemia	9(8.1)	1 (4.5)	8 (9.0)	0.685
Stroke	11 (9.9)	5 (22.7)	6 (6.7)	0.025
Smoking	41 (36.9)	12 (54.5)	29 (32.6)	0.056
Previous PCI	7 (6.3)	3 (13.6)	4 (4.5)	0.138
LVEF (%)	63.54 ± 0.6	62.46 ± 0.9	63.74 ± 0.1	0.347
Diagnoses				0.171
SAP	40 (36.0)	12 (54.5)	28 (31.5)	
UAP	59(53.2)	8 (36.4)	51 (57.3)	
STEMI	9(8.1)	2 (9.1)	7 (7.9)	
NSTEMI	3 (2.7)	0 (0)	3 (3.4)	
Invasive blood pressure				
SBP (mmHg)	1252 ± 0	1222 ± 1	1252 ± 0	0.500
DBP (mmHg)	821 ± 3	801 ± 5	821 ± 2	0.374

*PCI, percutaneous coronary intervention; LVEF, left ventricular ejection fraction; SAP, stable angina pectoris; UAP, unstable angina pectoris; STEMI, ST segment elevated myocardial infarction; NSTEMI, non-ST-segment elevated myocardial infarction; SBP, systolic blood pressure; DBP, diastolic blood pressure*.

Mean caFFR value in all lesions was 0.86 ± 0.09. Lesions with caFFR ≤ 0.8 showed lower MLA and higher AS compared with caFFR > 0.8 (MLA: 3.3 ± 0.8 vs. 4.1 ± 1.2, *P* = 0.002; AS: 71.3 ± 9.6% vs. 63.5 ± 1.3%, *P* = 0.007). Diameter stenosis (DS%) and lesion length (LL) evaluated by 3D-QCA were more severe in the group of caFFR ≤ 0.8 (DS%: 45.5 ± 9.6% vs. 35.5 ± 8.2%, *P* < 0.001; LL: 31.6 ± 12.9 vs. 21.0 ± 12.8, *P* < 0.001) (Table 2).

**Table 2:** Lesion characteristics between the caFFR ≤ 0.8 group and the caFFR > 0.8 group.

**Lesion characteristics**	**All*N* = 122**	**caFFR ≤ 0.8 *N* = 24**	**caFF > 0.8*N* = 98**	***P* value**
IVUS-parameters				
MLA (mm^2^)	4.01 ± 0.2	3.30 ± 0.8	4.11 ± 0.2	0.002
AS (%)	65.11 ± 0.3	71.39 ± 0.6	63.51 ± 0.3	0.007
3D-QCA parameters				
Diameter stenosis (%)	37.59 ± 0.4	45.59 ± 0.6	35.58 ± 0.2	<0.001
Lesion length (mm)	23.01 ± 3.5	31.61 ± 2.9	21.01 ± 2.8	<0.001
Flow speed (cm/s)	144.55 ± 2.7	159.34 ± 8.1	140.15 ± 3.3	0.126
Target vessels				1.000
LAD	97 (79.5)	19 (79.2)	78 (79.6)	
LCX	6 (4.9)	1 (4.2)	5 (5.1)	
RCA	19 (15.6)	4 (16.7)	15 (15.3)	
Treatment				0.082
Stenting	52 (42.6)	14 (58.3)	38 (38.8)	
Medical treatment only	70 (57.4)	10 (41.7)	60 (61.2)	
caFFR value	0.860 ± 0.09	0.720 ± 0.07	0.890 ± 0.05	<0.001

*IVUS, intravascular ultrasound; MLA, minimal lumen area; AS, area stenosis; 3D-QCA, three-dimensional quantitative coronary angiography; LAD, left anterior descending branch; LCX, left circumflex artery; RCA, right coronary artery*.

**Figure 2:**
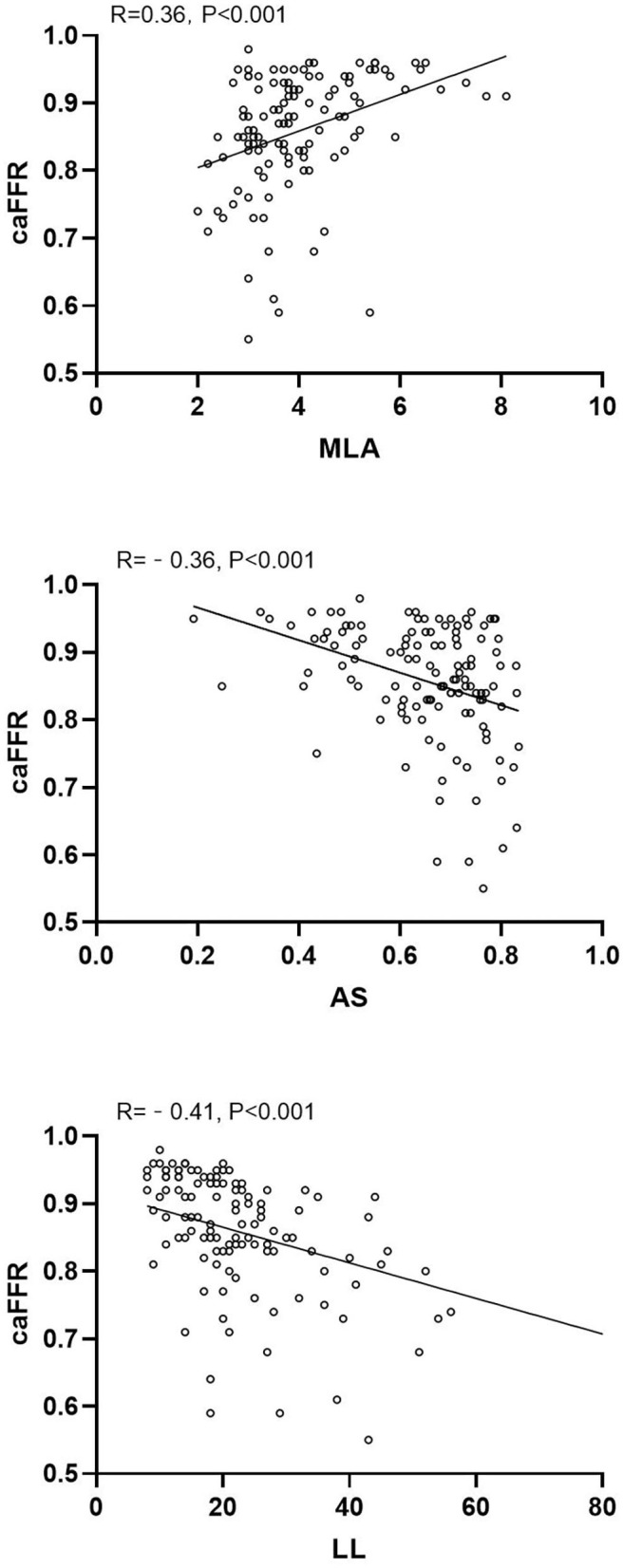
Correlation between caFFR and MLA, AS, and LL.

caFFR showed moderate correlations with MLA, AS, and LL (MLA: *r* = 0.36, *P* < 0.001; AS: *r* = −0.36, *P* < 0.001; LL: *r* = −0.41, *P* < 0.001) (Figure 2). Moreover, a multiple linear regression analysis demonstrated that MLA (β = 0.218, *P* = 0.013), AS (β = −0.197, *P* = 0.029), and LL (β = −0.306, *P* < 0.001) contributed significantly to the variation in caFFR (Table 3). When defined caFFR ≤ 0.8 as the threshold for myocardial ischemia, the best cutoff value of MLA to detect functional ICS was 3.6 mm^2^, with the area under the curve (AUC) of 0.714, sensitivity of 75%, specificity of 63%, positive predictive value (PPV) of 33%, and negative predictive value (NPV) of 91%. In addition, the best cutoff value of AS to identify functional ICS was 73%, with AUC, sensitivity, specificity, PPV, and NPV of 0.688, 54, 77, 36, and 87%, respectively. The diagnostic accuracy of LL to determine caFFR ≤ 0.8 was ideal (AUC 0.767, sensitivity 58%, specificity 82%, PPV 44%, NPV 89%, *P* < 0.001).

**Table 3:** Linear regression analysis of the effect of MLA, AS, and LL on caFFR.

	**Univariate analysis**			**Multivariate analysis**		
**Variables**	**β**	**95% CI**	***P* value**	**β**	**95% CI**	***P* value**
Intercept				0.924	0.818–1.013	<0.001
MLA	0.361	0.014–0.039	<0.001	0.218	0.004–0.029	0.013
AS	0.352	−0.311–−0.108	<0.001	−0.197	−0.249–−0.014	0.029
LL	−0.423	−0.003–−0.001	<0.001	−0.306	−0.003–−0.001	<0.001

*β, standardized regression coefficient; CI, confidence interval; MLA, minimal lumen area; AS, area stenosis; LL, lesion length*.

**Figure 3:**
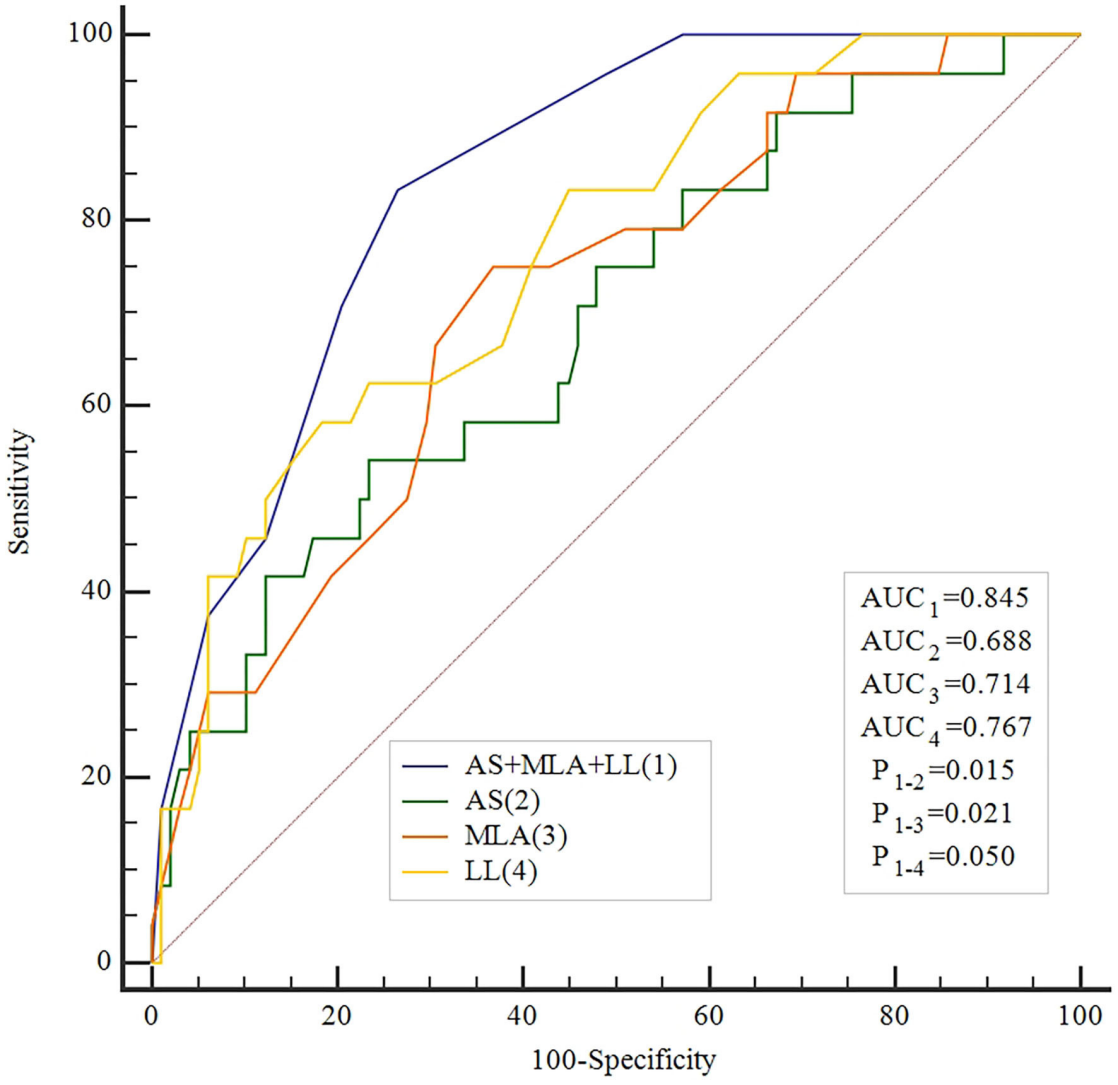
Receiving operator curve for caFFR and MLA, AS, LL, and their combination.

Moreover, when combined MLA, AS, and LL for identifying functional ICS, the accuracy was higher than all other single measures (AUC: 0.845, sensitivity 83%, specificity 73%, PPV 44%, NPV 95%, *P* < 0.001, Table 4). The ROC showed that the accuracy of the combined method was significantly higher compared with every single method alone (Figure 3).

**Table 4:** Diagnostic value of different variables.

**Index**	**Positive threshold**	**Area**	**Sen**	**Spe**	**PPV**	**NPV**	***P* value**
							
MLA	≤3.6	0.714	75	63	33	91	<0.001
AS	>73%	0.688	54	77	36	87	0.002
LL	>26	0.767	58	82	44	89	<0.001
MLA+AS+LL		0.845	83	73	44	95	<0.001

*MLA, minimal lumen area; AS, area stenosis; LL, lesion length; Sen, sensitivity; Spe, specificity; PPV, positive predictive value; NPV, negative predictive value*.

## Discussion

The present study correlated IVUS-parameters and LL assessed by 3D-QCA with caFFR in intermediate stenosis of non-LMCA. MLA, AS, and LL demonstrated a weak correlation with caFFR. We identified that MLA ≤3.6 mm^2^, AS >73%, and LL >26 mm as the highest diagnostic accuracy to reveal functional ICS confirmed by caFFR. The combination of these three parameters provides an incremental diagnostic accuracy to each measure separately.

In clinical practice, although IVUS examination is commonly used for assisting management strategies in ICS lesions, the cutoff value of the parameters for detecting functional ICS remained controversial. A previous study suggested that IVUS-MLA ≤ 4 mm^2^ was considered as functional stenosis which may require revascularization (12). Ben-Dor et al. identified an MLA < 3.6 mm^2^ as the best threshold value (AUC 0.70) in lesions with reference vessels diameter larger than 3.5 mm for FFR < 0.8 (1). Takagi et al. analyzed 51 lesions with coronary stenosis with both IVUS and FFR, concluding that an MLA < 3.0 mm^2^ and AS > 60% showed the highest accuracy (AUC 0.74) (13). In this study, we found that MLA ≤ 3.6 mm^2^ and AS >73% presented the best diagnostic power for predicting functional ischemia defined by caFFR (AUC 0.714 for MLA, AUC 0.688 for AS). The accuracy of IVUS parameters differed greatly among studies (1, 10, 12). On the other hand, MLA or AS is one of many factors that influence coronary flow, which only reflect a single dimensional anatomic change and could not reflect other factors such as the amount of segmental lesion (13).

Quantitative angiography-derived parameters correlated weakly to moderately with functional ICS. Kang et al. demonstrated a significant but weak correlation between LL and FFR in lesion with lumen area <3 mm^2^ (*r* = −0.47) (9). Naganuma et al. also showed a weak linear correlation between LL and FFR (*r* = −0.348) (14). In our study, LL was weakly correlated with caFFR, which was consistent with the previous study. Furthermore, our result showed LL > 26 mm powered a functional significance. However, Kang et al. demonstrated that the best cutoff value of LL for predicting FFR < 0.8 was 3.1 mm (9). In this study, the mean LL was only 4.6 ± 6.0 mm, which could explain the very low threshold of LL in functional lesions. Lopez-Palop et al. revealed that long lesions (>20 mm) with moderate angiographic stenosis might be one of the determinants of functional significance (11). Moreover, Koo studied the effect of lesion characteristics on the diagnostic performance of machine learning-based computed tomography-derived fractional flow reserve (ML-FFR), showing a downward trend of ML-FFR along with an increase in lesion length (15). Thus, we concluded that LL should be considered when judging the benefit of revascularization.

Although MLA, AS, and LL had a certain role in identifying functional ischemia, their diagnostic efficiency was still of concern. The FIRST study showed an AUC of 0.65 with an MLA < 3.07 mm^2^ to predict FFR < 0.8, which was a quite low diagnostic power (10). Moreover, Lopez-Palop et al. illustrated a better diagnostic power of LL in determining FFR < 0.8 (AUC 0.78) (11).

As an essential variable influencing the pressure difference between the proximal to the distal lesion, MLA subjected to the heterogeneity of the reference vessel diameter, and the effect of AS on blood flow should also be considered. Moreover, in the principle of fluid dynamics (16), LL is a crucial variable affecting the flow of the vessels. Therefore, a model combining the three indexes was constructed in our study to acquire higher accuracy.

After the combination of MLA, AS, and LL, the AUC elevated to 0.845, which is greater than each index measure alone. We present a high overall sensitivity of 83% showing a combined model could identify more positive lesions, but PPV is only 44%, indicating that revascularization should be under cautious consideration when the combined model shows positive results. In this case, physicians need to obtain more information, like FFR or radionuclide myocardial perfusion. Furthermore, the specificity of the combined model is passable, but the NPV is as high as 95% allowing revascularization to be deferred with greater confidence when MLA is larger, AS is lower, and/or LL is short enough.

In summary, although anatomic features assessed by IVUS or 3D-QCA have a certain role in predicting myocardial ischemia, their accuracy was unsatisfactory in clinical practice. By integrated MLA, AS, and LL to evaluate overall diagnostic power, a combined method provides higher accuracy than each measure alone.

## Limitations

There are several limitations of this study. First, this is a single center, retrospective study. Although we consecutively screened eligible patients, around 43.5% of them were excluded, selective bias was inevitable. Second, the number of analyzed vessels from LCX or RCA was insufficient. Finally, caFFR values were assessed offline, aortic pressure was input with the invasive blood pressure reviewed from the record of operation. A large multi-center prospective cohort study with a larger number of LCX and RCA should be conducted.

## Data Availability Statement

The raw data supporting the conclusions of this article will be made available by the authors, without undue reservation.

## Ethics Statement

The studies involving human participants were reviewed and approved by the Independent Institutional Ethical Committee of The First Affiliated Hospital of Nanjing Medical University. The patients/participants provided their written informed consent to participate in this study.

## Author Contributions

ML: conceptualization, methodology, investigation, formal analysis, and writing—original draft. XK: validation and supervision. HW: conceptualization and investigation. YH and IC: writing—review and editing. SL: formal analysis. All authors contributed to the article and approved the submitted version.

## Funding

This paper was funded by Science Foundation of Gusu School (GSKY20210105) and Natural Science Foundation of Jiangsu Province (BK2012648 to HW). The funding body played no role in the design, writing, or decision to publish this paper.

## Conflict of Interest

The authors declare that the research was conducted in the absence of any commercial or financial relationships that could be construed as a potential conflict of interest.

## Publisher's Note

All claims expressed in this article are solely those of the authors and do not necessarily represent those of their affiliated organizations, or those of the publisher, the editors and the reviewers. Any product that may be evaluated in this article, or claim that may be made by its manufacturer, is not guaranteed or endorsed by the publisher.
